# Utilization of Hydroxyl-Methyl Butyrate, Leucine, Glutamine and Arginine Supplementation in Nutritional Management of Sarcopenia—Implications and Clinical Considerations for Type 2 Diabetes Mellitus Risk Modulation

**DOI:** 10.3390/jpm10010019

**Published:** 2020-03-24

**Authors:** Adeline Maykish, Angelos K. Sikalidis

**Affiliations:** Department of Food Science and Nutrition, California Polytechnic State University, San Luis Obispo, CA 93407, USA; amaykish@calpoly.edu

**Keywords:** sarcopenia, hydroxyl-methyl butyrate, leucine, glutamine, arginine, T2DM

## Abstract

While onset characteristics may vary, sarcopenia gradually develops over time as a result of the aging process, leading to muscle loss, disturbance of the muscle to fat ratio, and a variety of negative symptoms undermining the wellbeing, quality of life, and lifespan in the aging population globally. There is evidence that sarcopenia may be a cause and consequence of type 2 diabetes mellitus (T2DM) in the aging population. The importance of nutritional management in the prevention and/or deceleration of sarcopenia is critical, with the main focus placed on the amount and quality of protein intake. Significant efforts are being made towards the development of medical nutrition therapies involving certain amino acids and amino compounds, as well as their combinations, for the improvement in muscle strength, muscle function and protein synthesis. This may reduce hospitalization times and hasten the recovery of patients with sarcopenia. The administration of protocols with varying dose and frequencies, as well as their efficacy, is being investigated. In the work herein, we present and evaluate data derived from human trials regarding the utilization of hydroxyl-methyl butyrate (HMB), L-leucine (Leu), L-glutamine (Gln) and L-arginine (Arg) supplementation for optimal management of sarcopenia in geriatric patients, a topic of significant clinical nutrition interest which may have important implications in T2DM status.

## 1. Introduction

Aging, defined as the progression of deterioration with diminishing life functions gradually leading to the end of life, is a global phenomenon of significant study [1]. The elderly, defined by the World Health Organization (WHO) as individuals 65 years of age or older, exhibit an annual population increase of 5% in both developed and developing countries [2,3]. One of the various effects of aging is the progressive loss of muscle mass over time. Muscle mass, which accounts for approximately one-third of the total body mass/weight in the youth, has been shown to deteriorate with age. Muscle strength is reduced by 8%–10% per decade while an average of 5% of actual mass is lost every decade after the age of 40 [4,5,6,7]. This age-related progressive loss of muscle mass is termed sarcopenia (derived from the Greek σαρξ (sarc): “of flesh”, and πενία (penia): “poverty”, hence sarcopenia literally meaning: “poverty of the flesh/muscle”). The term sarcopenia was first introduced in 1989 by Irwin Rosenberg, who commented that: “no decline with age is more dramatic and potentially more functionally significant than the decline in lean body mass” [8]. Sarcopenia is a geriatric syndrome typically accompanied by a decrease in physical activity and performance function, as well as protein–energy malnutrition [9,10]. 

Commonly, sarcopenic patients exhibit muscle fiber reduction, muscle atrophy [11], and reduced oxygen consumption due to decreased muscle mass without changes in average body weight. In fact, body weight may increase due to the increase in body fat [1]. For this reason, sarcopenia is observed not only in low body mass index (BMI) in relation to general malnutrition and/or protein-energy malnutrition, but also in cases where fat to muscle ratio increases [12]. Due to structural and functional tension in the muscles of sarcopenic patients, there is an increase in mobility restriction, fragility, falls and morbidity, which, in turn, disrupts the body’s metabolic adaptation to stress and illness [11,13]. While the effects of sarcopenia on muscle function-related aspects are broadly appreciated, the metabolic consequences receive less attention even though they are equally significant and potentially more challenging to control clinically. Specifically, as lean body mass is reduced, several pathways responsible for inducing insulin resistance, ultimately leading to type 2 diabetes mellitus (T2DM), are promoted [14]. Considering that 80% of glucose clearance is achieved by muscle tissue, under euglycemic conditions [15], it can be deduced that a decline in muscle mass and/or quality, may directly and dramatically interfere with glucose clearance capacity, thus leading to hyperglycemic episodes and increased T2DM risk. Improving sarcopenic status may prove critical not only for the muscle functional considerations but also as a means of T2DM prevention and/or management.

Typically, with sarcopenic patients, the major goal of medical nutrition therapy is to prevent/delay further muscle decline, improve muscle mass quality, as well as support immunity and wound healing. Studies with such patients have evaluated the effectiveness of nutritional supplementation in the clinical setting with very limited evidence, suggesting that there may be some positive effects on muscle mass quality and function through amino acid supplementation [15]. In a 2018 study, 68 individuals aged 70 and older were evaluated for their amino acid profile. Of these participants, 38 had physical frailty and sarcopenia, and 30 non-sarcopenic, non-frail individuals served as controls. It was determined that the sarcopenic individuals had higher serum levels of asparagine, aspartic acid, citrulline, ethanolamine, glutamic acid, sarcosine, and taurine. The non-sarcopenic individuals, on the other hand, displayed elevated amounts of α-aminobutyric acid and methionine only [16]. This provides some insight as to what amino acids truly are necessary for supplementation, and which are the most beneficial for muscle synthesis and/or protection against degradation and loss. Since amino acids are sourced from protein, increased dietary intake of high-quality protein might be an effective mode for stimulating muscle protein synthesis and promoting gains in muscle mass, strength and function, while further enhancing exercise-induced physiological adaptations [17], although whether such practice would suffice is unclear. 

Among the four nutritional substrates that affect muscle proteolysis, hydroxyl-methyl butyrate (HMB) is an active metabolic form of leucine (Leu); Leu, along with glutamine (Gln) are essential amino acids (EAA), while arginine (Arg) is a semi-essential amino acid (SEAA). All of the aforementioned compounds extend anti-catabolic and anabolic stimuli for muscle synthesis, performance and immune system improvement [17,18]. Further, Gln is one of the most abundant amino acids that may become essential in critical conditions [19]. A mixture of HMB, Leu, Gln and Arg was recently evaluated in the context of nutritional status, quality of life, treatment side effects, serum parameters, anthropometric data, and bioavailability in elderly patients undergoing cardiac surgery [20]. HMB, Gln and Arg were shown to be effective in slowing muscle loss and reducing circulating levels of muscle protein amino acids [20]. 

The work presented investigates and discusses the limited available evidence regarding strategies of supplementation, including amino acids. Although not commonly used, such strategies show promise for the nutritional management of sarcopenia with potential implications for T2DM.

## 2. General Considerations for HMB, Gln, Arg, Leu

Several *in vivo* and human studies have shown that HMB extends a notable anticatabolic effect on skeletal muscle by minimizing muscle damage and muscle proteolysis [21]. Although the mechanisms of HMB contributing to the reduction in muscle protein degradation are not fully understood, it is thought that HMB affects muscle mass in its own right, independently of Arg and Gln [21]. HMB, in conjunction with Arg and Gln, improves nitrogen metabolism in critically ill patients, increases protein synthesis over prolonged use and has been seen to reduce protein degradation, thereby effectively defending lean body mass [22,23].

Leucine is an essential dietary amino acid that confers stimulatory signaling for muscle protein synthesis [24]. It is closely linked to the release of alanine from muscles, whereby alanine functions as a gluconeogenic precursor, thus inducing gluconeogenesis, a process that allows glucose to be synthesized from non-carbohydrate precursors, allowing for glucose homeostasis as needed in the absence or limited presence of dietary carbohydrates [25].

Glutamine is one of the most abundant amino acids in the body, produced mostly in skeletal muscle and metabolized by the intestine, kidney and liver [19]. While not considered an essential amino acid in homeostatic conditions, Gln can become essential in critical conditions such as cachexia [19]. 

Arginine is a semi-essential amino acid and a precursor to nitric oxide, a major signaling molecule and vasorelaxant [26]. Further, Arg plays a pivotal role especially in critical illnesses and serious trauma as it promotes the secretion of various hormones in conditions of stress and sepsis [27,28].

While the mechanisms of age-related loss of muscle mass are not completely understood, Arg and Gln support enhanced net protein synthesis, and their co-administration with HMB further reduces protein breakdown [23] seemingly maximizing the maintenance of total lean body mass. 

Appropriate dietary interventions providing positive nitrogen balance can reduce muscle loss and induce anabolic effects in sarcopenia [29].

### 2.1. Supplementation with HMB

Hsieh et al. [30] examined 34 elderly patients (78–79 years old) with chronic obstructive pulmonary disease (COPD) in an intensive care setting. The patients required mechanical ventilators and demonstrated muscle wasting. They were randomly assigned to HMB (*n* = 18) or control (*n* = 16) groups, where the HMB group received 3 g HMB daily for one week. HMB supplementation extended anti-inflammatory and anticatabolic effects, as TNF-α levels were significantly reduced in participants on HMB, while body weight did not change in either group.

In a separate study by Hsieh et al. [22], the effect of HMB supplementation on body composition and protein metabolism was investigated during tube-feeding to bedfast elderly in a nursing home setting. The participants were divided into two groups: an HMB (*n* = 39, at 2 g/day) and a control (*n* = 40) group. Body weight and changes in BMI were not significantly different between groups after 14 or 28 days. Blood urea nitrogen (BUN) was decreased significantly in the HMB group but remained unchanged in the control group after 14 days. Urinary urea nitrogen (UUN) excretion decreased significantly in the HMB group, whereas the control group demonstrated a significant increase in 14 and 28 days. After controlling for the baseline BMI, changes in BUN and UUN excretion were significantly different between the groups and consistently lower for the HMB group. Although BMI was not changed in the control group, body weight and BMI showed small yet statistically significant increases after 28 days in the case of the HMB group. This work showed that HMB support for 2–4 weeks can reduce muscle degradation in elderly residents in an inpatient unit setting.

Additionally, Duque et al. [31] compiled a meta-analysis to determine the effect of HMB on muscle mass, strength, and function regarding sarcopenia and frailty. Three matching studies were found, totaling 203 patients. These patients demonstrated an increase in lean muscle mass, muscle strength and function after HMB supplementation, and it was concluded that HMB supplementation can increase muscle strength in elderly individuals and sarcopenic patients.

### 2.2. Supplementation with Leu

Katsanos et al. [32] investigated the effect of Leu in the context of enrichment of an EAA formula supplement, and its effect on muscle protein synthesis in young and elderly participants. Two elderly and two young groups were assessed before and after ingestion of 6.7 g of EAA formula. Formula was composed of whey protein (26% Leu) or enriched in Leu (41% Leu). In the elderly, the Leu-enriched EAA mixture stimulated postprandial muscle protein synthesis and accretion of muscle proteins, in contrast to the lack of response following the whey protein-based EAA mixture. The study provided evidence that a relatively small bolus of ingested Leu (≈3 g) in elderly individuals can acutely improve muscle protein retention and reverse a lack of stimulation of muscle protein synthesis. Their work emphasized the importance of L-Leu in any formulation of any amino acid/protein supplement and the effectiveness of such nutritional supplementation in reversing the attenuated response of muscle protein synthesis in the elderly, thus salvaging the healthy muscle phenotype, at least partially.

Similarly, Koopman et al. [33] studied the effects of carbohydrate, or carbohydrate plus protein and free Leu, as a means to evaluate the changes in muscle protein metabolism with age. Eight elderly (75 ± 1 year) and eight young (20 ± 1 year) consumed, either carbohydrate or carbohydrate plus protein and free Leu after completing 30 minutes of standard daily activities. Carbohydrate was provided to prevent a negative protein balance. Blood and muscle samples were then assessed to determine protein turnover. In both groups, the Leu supplementation showed improved protein balance compared to the carbohydrate group, while muscle protein synthesis rates were also greater in the supplemented group. This means that Leu supplementation is beneficial regardless of age, and it may even be advantageous to start supplementing at a young age to decrease risk [33,34].

Muscle protein synthesis is shown to be responsive to Leu supplementation in older rats [35]. Adult (8 months) and old (22 months) rats were fed an 18.2% protein semiliquid control diet for one month. On the day of assessment, rats received either no food (post-absorptive group), or an Ala- or Leu-supplemented meal for 1 hour (postprandial group). The *de novo* muscle protein synthesis, assessed 90–120 minutes after the meal distribution, was significantly greater in the adult postprandial group, whereas it was not stimulated in the old comparable group. However, when Leu supplementation occurred, muscle protein synthesis in old rats was stimulated at similar levels to that observed in adults, indicating that Leu can salvage the metabolic phenotype in older animals, at least in an acute phase [35].

Rieu et al. [35] further reported in older rats that the muscle protein synthesis following a normal mixed nutrient meal was blunt compared to younger animals. In elderly participants, the addition of Leu to a mixed food meal led to a meaningful improvement in muscle protein synthesis, regardless of an overall increase in other amino acids [36]. Interestingly, using adult growing rats, Debras et al. [37] demonstrated that postprandial Leu deficiency failed to alter muscle protein synthesis in growing and adult rats, indicating that the supplementation alone is not the sole determinant for muscle protein synthesis as age and that the amino acid pool also needs to be considered. The addition of supplemental dietary Leu, however, increased the expression levels of muscle proteins in older rats compared to that of young [37].

Whey protein is commonly used as a post-workout dietary supplement, as it is believed to aid in the building of muscle mass, partially due to the fact that it is a good source of Leu. In a 2012 study, 37 elderly men (71 ± 4 years) were provided whey protein supplement to investigate the potential increase in muscle protein synthesis. The participants completed leg-based resistance exercises, then ingested 0, 10, 20, or 40 g of whey protein isolate, W0 to W40, respectively. Muscle protein synthesis increased 65% for W20 and 90% for W40, while lower doses elicited no change in muscle protein synthesis [38]. Notably, another study [32] also reported that younger adults needed only 40 g to saturate muscle protein synthesis compared to older counterparts. This suggests that whey protein is a valuable source of Leu and does aid in muscle protein synthesis, but elderly individuals should be aware that a higher dose is needed to get the true benefits of the supplement. Whey protein is a rather accessible dietary supplement due to its availability at most grocery stores and could be recommended to elderly patients as a potential means to manage sarcopenia.

Similarly, a recent study was carried out involving 49 elderly men (73 ± 1 year) who consumed a nutritional supplement (SUPP) consisting of whey protein, creatine, calcium, vitamin D, and n-3 polyunsaturated fatty acid (PUFA), or a control (CON) consisting only of maltodextrin. Phase one of this study consisted only of twice-daily dietary supplement, while phase two consisted of twice-daily dietary supplement in conjunction with exercise three times per week. During phase one, the SUPP group displayed increased strength, but not the CON group. Both SUPP and CON groups gained strength during phase two, but the SUPP group gained significantly more muscle strength and lean muscle mass while demonstrating improved metabolic health, as well as larger aerobic capacity. A glucose tolerance test was also performed, where, interestingly, the maximal glucose concentrations decreased in the supplemented patients, highlighting the link between muscle strength and glucose management. The increase in lean muscle mass and muscle strength during phase one in the SUPP patients suggests that whey protein supplement is beneficial for elderly individuals as a means of sarcopenia treatment in the absence of exercise [39].

In a randomized study by Solerte et al. [40], 41 people with sarcopenia between the ages of 66 and 84 years were provided 8 g EAA for 18 months. Increased muscle mass, insulin sensitivity, and insulin-like growth factor 1 (IGF-1) were observed as a result of supplementation, as well as decreased tumor necrosis factor alpha (TNFα). In separate experiments conducted by the same group of researchers, improvement in glycemia control and insulin sensitivity was reported during a long-term (60-week) randomized study with amino acid dietary supplements in elderly participants diagnosed with T2DM. These findings support the recommendation of using a balanced amino acid supplement enriched with Leu to slow muscle loss at least in the elderly and/or chronically diseased population, especially in the case of T2DM. The addition of 2 g HMB per day to bedridden elderly in nursing homes was shown to reduce muscle wasting significantly [30], and when a cocktail of 3g HMB + 7.5g Arg + 2.25g lysine (Lys) was given to elderly men and women, protein turnover and lean tissue mass increased [26]. Obtained results persisted at the end of the one-year period assessed, suggesting potential for long-term, lasting, positive effects in agreement with other observations. Additionally, Ferrando et al. [41] reported that a supplementation of EAAs (15 g, 3 × per day) to bedrest-immobilized elderly subjects, achieved the maintenance of the 24-hour fractional synthetic rate (FSR), an index of muscle protein synthesis. Other groups showed a positive effect on quality of life indices as well as amino acid profile and strength in institutionalized elderly supplemented with amino acids, including Leu [42]. Furthermore, in a systematic review and meta-analysis, Leu supplementation was determined to be effective on muscle protein synthesis assessed by FSR, without affecting lean body mass or leg lean mass accretion in elderly patients participating in nine randomized controlled trials. Hence, such therapeutic schemes may be beneficial for elderly with sarcopenia [43]. Recently, Murphy et al. documented that, in healthy old subjects, Leu supplementation increased the integrated muscle protein synthesis response [44]. In contrast, the relationship between Leu supplementation and improved muscle protein synthesis was not verified in human trials involving young men. More specifically, Leu supplementation was not shown to attenuate skeletal muscle loss during leg immobilization in healthy young men [45]. 

Even though there are a variety of studies that have shown that Leu supplementation can increase protein synthesis, it remains unclear what dosing and frequency regime would be optimal in terms of efficiency and sustaining results, especially when the conditions relative to age and health status are factored in. For example, several reports revealed that muscle atrophy during short-term (28 days) and long-term bedrest (60 days) failed to be impacted by daily amino acid supplementation, or by a daily Leu-enriched whey protein supplement, respectively [46,47,48]. At the same time, there is a long-standing concern regarding the ability to positively influence muscle mass while age progresses, since muscle may gradually develop more resistance to the stimulatory effects of normal postprandial Leu concentrations [33]. Although such a deficiency contributes to the lowering of muscle protein anabolism and loss of muscle mass, according to a systematic review and meta-analysis of 16 studies, [49] supplemental Leu may result in aging muscle protein synthesis normalization or improvement, with no significant benefits regarding muscle strength. Long-term studies are still lacking, and final conclusions cannot be solidly drawn yet from the available scientific evidence. 

Even though free Leu supplementation acutely increases muscle protein synthesis, and muscle mass in some long-term studies, other studies have reported no increases in muscle mass following prolonged Leu supplementation [50]. Therefore, it appears that chronically increasing Leu intake via the consumption of an overall increase in dietary protein appears to be the most effective dietary intervention toward increasing or attenuating lean mass during aging. However, more research investigating the optimal dose and timing of protein ingestion is necessary. Several studies have demonstrated that decreases in postprandial muscle protein synthesis as a result of increased circulating oxidative and inflammatory markers contribute more than muscle protein breakdown to the decreases in muscle mass during disease and healthy aging [50]. Dietary interventions reducing oxidative/inflammatory stress combined with higher protein quality, and intake amounts able to overcome anabolic resistance, may enhance the muscle protein synthesis response to feeding, and either increase muscle mass or attenuate loss depending on the case. Nonetheless, it remains unclear as to why chronic Leu supplementation, despite its powerful effects on acute muscle protein synthesis, only sometimes translates into increased muscle mass when evaluated chronically. 

### 2.3. Supplementation with Arg

The effect of a nutritional supplement containing essential amino acids (EAA)+Arg was determined in 12 elderly participants (67.0 ± 5.6 years, seven females, five males) who were glucose intolerant. For 16 weeks, 11 g of EAA+Arg was administered twice daily, between meals, while diet and activity were not otherwise changed. During the study, lean body mass (LBM) increased by 1.14 ± 0.36 kg at week 12, and 0.60 ± 0.38 kg at week 16. An improvement was noted for other parameters, including lower extremity strength measure score and usual gait speed, as well as timed five-step and floor transfer tests. The authors concluded that dietary supplementation with EAA+Arg increased fat-free body mass, strength and physical function in elderly individuals with glucose intolerance [51].

A sample of 21 elderly individuals, with an average age of 68 years, deemed to be moderately active, participated in a 2010 study. The goal was to determine how supplementation with various EAAs affects muscle mass and function after 10 days in a bedrest regime. This model was chosen because elderly individuals comprise the majority of hospitalized individuals and report a decreased ability to complete relative daily activities (RDA) after hospitalization. Specifically, 10 were provided 15 g of EAA, consisting primarily of Leu and Lys, followed by Arg, while 11 of the participants were provided a placebo (control). The fractional synthetic rate was maintained in the EAA group, while it decreased by 30% in the control. However, when this was adjusted for pre-bedrest value and sex, there was no significant difference. There was also no effect of EAA on leg muscle mass maintenance, nor fat mass. Notably, the study design did not include an exercise regime, unlike several other studies. Hence, EAA supplementation alone is likely not adequate to prevent muscle deterioration in the elderly. Supplementation along with appropriate exercise seems to be more beneficial [52].

Chronic kidney disease (CKD), leading to Arg deficiency, is often associated with sarcopenia. The effects of Arg supplementation, in combination with resistance training exercises, in rats was evaluated to determine the effectiveness of the treatment after 10 weeks. Specifically, 25 eight-week-old rats were grouped into control sedentary, CKD sedentary, CKD sedentary with 2% Arg supplementation, CKD with exercise, and CKD with both exercise and supplementation. It was reported that supplementation alone was not adequate to manage CKD, and affect muscle mass, or inflammation. Interestingly, the combination of resistance training and supplementation fared worse than just resistance training. It is also speculated that Arg supplementation provided an excess of nitrogen, deteriorating renal function and leading to an increase in reactive oxygen species. Therefore, Arg supplementation does not appear to be beneficial in CDK [53].

The effect of a combination supplement of HMB, Arg, and Leu was evaluated in 50 elderly women for 12 weeks. Strength was measured using the “get-up-and-go” test, where someone starts at a seated position, stands, walks 3 m, turns around, and returns to the seat. At the end of the 12 weeks, the supplemented groups showed a 17% improvement in this test, whereas the placebo group demonstrated a decrease in performance. The supplemented group also exhibited an increase in knee flexor force, knee extensor force, and handgrip strength, all supporting the belief that muscle quality improves with supplementation. Further, in the supplemented group, whole body protein synthesis was about 20% greater than in the placebo group. Thus, supplementation combining HMB, Arg and Leu seems to provide adequate protection against muscle deterioration. HMB is known to slow protein breakdown, and Arg and Leu support protein synthesis; therefore, a combined supplement can potentially decline the loss of muscle mass that leads to sarcopenia [54]. While Arg alone does not appear to be sufficient to prevent muscle deterioration, when combined with HMB and Leu it does seem to be effective. 

### 2.4. Supplementation with Gln

Sarcopenia is a common outcome of gastrectomy surgery, as alimentary function is disrupted and nutrient uptake becomes inefficient. Glutamine, playing an important role in the signaling pathways of protein synthesis and protein degradation, was investigated as a means of dietary supplementation in rats after total gastrectomy. Rats were divided into five groups: sham, gastrectomized only, gastrectomized with branched chain amino acid (BCAA) supplementation, gastrectomized with Gln supplementation, and gastrectomized with both BCAA and Gln. In the BCAA/Gln group, weight gain was significantly increased in comparison to the gastrectomized only. Additionally, this combination inhibited muscle atrophy more than either one or no supplementation. Therefore, Gln in combination with BCAA supplementation (such as Leu) may be vital for sarcopenia prevention [55]. 

In another study with rats, Gln supplementation was investigated as a means of upregulation of Gln synthetase (GS). Glutamine synthetase activity was analyzed after 5 days of fasting in adult and very old female rats. The rats were also analyzed in their re-fed state with supplementation of Gln, alanine (Ala), or glycine (Gly). After the 5 days of fasting, regardless of whether supplementation was Gln, Ala, or Gly, GS activity was decreased in adult rats. However, in the very old rats, decreased GS activity was only seen with Gln supplementation, with no effect of Gln supplementation observed in the fed state. In the very old rats, intramuscular Gln was depleted, resulting in the observed decrease in GS activity, in agreement with previous evidence indicating that sarcopenia is often associated with malnourishment [56].

While glutamine supplementation initiated in rats before advanced age can improve gut mass and function in advanced age, it does not seem to prevent muscle wasting associated with age-related sarcopenia [57]. However, when combined with other amino acids, such as BCAA, Gln may show promise in preventing muscle degradation and sarcopenia.

### 2.5. Supplementation with HMB/Arg/Gln

Tatti and Barber investigated the effects of nutritional support on wound healing in patients with recurrent diabetic foot ulcers. Twelve patients received diets supplemented with 14 g Arg, 3 g HMB and 14 g Gln in two doses. Although there was no difference in total blood count, serum lipid biochemistry or hemoglobin A1c (HbA1c) values after the nutritional support, the duration of ulcer healing was significantly accelerated with supplementation. An improvement in lean body mass was also observed. The authors concluded that nutritional support with an HMB/Arg/Gln combination shortens the healing period of diabetic foot ulcers and increases lean body mass [58].

In a 24-week study, the effects of HMB, Arg, and Gln combination were evaluated in the prevention and reversal of cachexia on 49 advanced cancer patients (stage four) with documented weight loss (>5%). The patients were divided into two groups; one received daily supplementation of the mix: HMB (3 g), Arg (14 g) and Gln (14 g). The other group served as control and was given isocaloric and isonitrogenous product supplementation. At week four, the mix-supplemented group exhibited an increase both in body weight and lean mass. At week 24, the mix-supplemented group continued to increase in fat-free mass (FFM). The HMB/Arg/Gln combination was shown to be effective in increasing weight and FFM in advanced cancer patients with cachexia, was well tolerated, and no adverse events were reported [23].

HIV-infected patients with AIDS-related cachexia (*n* = 68) were given a nutrient mixture containing 3 g HMB, 14 g L-Gln, and 14 g L-Arg for 8 weeks in two doses daily. The participants administered the HMB/Arg/Gln mixture increased their weight by 3.0 ± 0.5 Kg, while those placebo-supplemented gained 0.37 ± 0.84 Kg at 8 weeks. The HMB/Arg/Gln-supplemented group had increased LBM (2.55 ± 0.75 Kg), whereas the placebo group lost lean mass (0.70 ± 0.69 Kg). The data indicate that the HMB/Arg/Gln mixture can significantly impact the course of lean tissue loss in patients with AIDS-associated wasting [21].

The long-term effects of daily amino acid supplementation on age-associated protein turnover and fat free mass changes in elderly participants (age 76 ± 1.6 years, 39 female and 38 male) were investigated in a double-blinded study. Study participants were randomly assigned to either supplement (HMB/Arg/Lys) (*n* = 40) or an isonitrogenous supplement serving as control (*n* = 37). At the end of the study, the HMB/Arg/Lys supplemented group increased their lean body mass tissue, while participants on the isonitrogenous supplement (control) demonstrated no change in lean body mass. Additionally, the HMB/Arg/Lys group demonstrated enhanced body cell mass by 1.6% (assessed by BIA) and lean mass by 1.2% (assessed via DXA) [59]. 

### 2.6. Supplementation with Leu/Arg/Gln

In 32 patients consuming a protein-rich enteral diet for 6 months in a hospital setting, no significant difference in anthropometric measurements was observed at the end of the study. As a result of the enteral diet consisting of BCCA (Gln and Arg at high levels), the essential amino acids in plasma, especially Leu, were increased while cortisol and 3-methylhistidine were reduced. These observations imply that protein synthesis signaling was enhanced, while protein degradation signaling was attenuated [60].

While there is no single supplement that can effectively treat sarcopenia, it appears that co-supplementation with the discussed amino acids may address the major issues associated with sarcopenia, creating a more promising course of treatment through synergistic benefits (Table 1). 

## 3. Discussion

According to the European Working Group on Sarcopenia (EWGSOP) report, there is a risk of sarcopenia in individuals who display muscle mass and/or muscle function loss [61]. Current studies in the literature indicate that additional substrates including HMB, Arg, Leu and Gln affect muscle proteolysis. The report revealed that each of them, when used individually or in combination, slows muscle loss and muscle protein turnover, which is effective in increasing lean body mass /muscle weight. Approaches including patient history, physical examination, anthropometric measurements, biochemical parameters (albumin, prealbumin), protein balance, and nutritional evaluation tests (BIA, SGA, NRS) were used in the included studies to evaluate patient nutrition status [61].

### 3.1. HMB

HMB, the active metabolite of Leu, when used alone, has been shown to have an anti-inflammatory effect and reduce TNF-α [30]. Stimulant effects were observed in the use of HMB alone, in patients with conditions causing muscle wasting (in elderly patients, such as those with AIDS, bedrest, cancer or neurodegenerative pathologies), and during periods of caloric deficiency [17]. Based on these results, it is suggested that HMB actually reduces the risk of both tumor formation and sarcopenia, and it is recommended that patients with risk should be supplemented daily. HMB, especially with physical activity support in patients, has been shown to increase muscle power and LBM, and to decrease the biochemical expression that induces muscle destruction, without side effects. The observed changes in body composition occurred with no changes in body weight. The simultaneous LBM and body fat loss in these patients lead to an improved lean-to-fat tissue ratio and, in turn, to improved body composition [62,63]. Despite sufficient evidence that lean body mass and strength gains are increased with the support of HMB, especially when combined with resistance exercise, no clear optimal dosage is suggested in the literature. The most common recommendation is to use 2–3 g/day (or 38 mg/Kg/day) [17,21,22,26,30], and in a variety of studies, 3 g HMB per day was the most beneficial dosage, without any observed side effects.

### 3.2. Leu

In animal studies, Leu-supplemented meals showed that the capacity to regain postprandial muscle protein synthesis persisted after a period of 10 days [35,64,65]. In elderly participants, a relatively small bolus of ingested Leu (3 g) indicates that muscle protein can increase retention and reverse muscle protein synthesis after a small amount of EAA support. Further investigation is needed to determine the duration and level of Leu reinforcement required to achieve muscle protein gain without negative effects. In humans, the use of Leu alone resulted in increased levels of plasma Leu, ultimately leading to a disproportionate balance for other amino acids. There is no evidence from long-term exposures to high doses of Leu as to how it may affect lean leg mass [43]. Currently, it is not recommended to use Leu alone, especially in long-term elderly supplementation. In the elderly, it is suggested that Leu should be used in combination with other essential amino acids on a long-term basis in order to increase the weakened response to protein support and the rate of protein synthesis.

### 3.3. HMB and Leu

Studies have indicated that HMB mediates the effects of Leu, stimulating mitochondrial biogenesis and effectively enhancing overall mitochondrial function. Being a Leu metabolite, HMB has a comparable impact, through protein synthesis mechanistic pathways involving mammalian Target of Rapamycin (mTOR), on mitochondrial biogenesis, leading to upregulation of relevant genes and increased mitochondrial mass. HMB supplementation is shown to increase β-oxidation (lipid utilization) at levels similar to those observed with Leu supplementation [66]. Conclusively, HMB appears to have similar effects on protein growth and the maintenance of lean body mass to those of Leu. Therefore, co-administration of Leu and HMB may, through synergistic action, potentially maximize benefits towards mitochondrial activation and defense of muscle mass. 

### 3.4. Arg—Arg and Gln

When Arg was used in combination with essential amino acids to supplement elderly patients, a significant increase was observed in LBM in the short term, while the positive effects did not persist in the long term [41]. Arg and Gln supplements are recommended as supplements in order to provide amino acid balance or to increase HMB–Leu activity in the treatments used [52].

### 3.5. HMB, Gln and Arg

In a study of collagen accumulation in wounds, the oral administration of a HMB/Gln/Arg mixture to elderly patients led to a significant increase in collagen synthesis [28]. When the same combination of effects on diabetic foot ulcers was investigated, the healing period required was significantly shortened [58]. Based on these results, use of an HMB/Gln/Arg combination in the treatment of complicated medical wounds may be recommended, as it induces healing in a side effect-free manner. When the same HMB/Gln/Arg mixture was used in both healthy elderly and patients with age-related pathologies (malnutrition, cancer, cachexia, AIDS), body weight and lean mass increased [22,67]. Overall, the use of this mixture confers improvements to patients in terms of hematological parameters (an increase in albumin and prealbumin levels), general healing, oxygen transport, immune response, and skeletal muscle mass in patients with lean tissue loss. Furthermore, supplementation with a mixture of HMB 2 g, Arg 5 g and Lys 1.5 g improved limb resistance, limb circumference and increased handgrip [54,68,69]. It also improved physical performance, muscle strength, FFM and protein synthesis.

The utilization of nutritional support mixtures leading to clinical picture improvements in nutritional parameters, especially for increasing albumin-prealbumin concentrations, are effective in malnutrition management and attract interest from clinicians. When mixtures containing essential, semi-essential and branched chain amino acids are used, blood plasma amino acid levels increase (especially Leu), while 3-methylhistidine and cortisol are decreased [43]. Decreases in cortisol and 3-methylhistidine indicate decreased protein degradation signaling in muscle. Therefore, the combination of EAA, SEAA and BCAA does not cause the amino acid imbalances seen in single uses. Since amino acid balance is not impaired, other amino acids can be produced and contribute to muscle proteolysis, therefore adequately supporting muscle turnover, while providing the signaling and building units for muscle mass deposition. Although a considerable consensus has been reached on the use of these supplement products for the treatment of sarcopenia, the search for optimal dosing, handling and exercise support of each amino acid supplement is ongoing.

## 4. Concurrent Therapies for Type 2 Diabetes Mellitus and Sarcopenia

**Figure 1:**
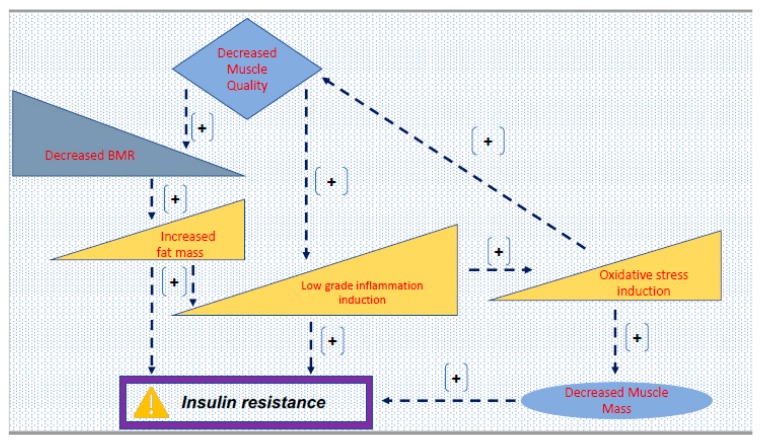
Potential pathways via which sarcopenia contributes to insulin resistance. [+] signs indicate an induction in the process (biochemical pathway), all eventually leading to stimulation of insulin resistance N.B.: Triangular shapes (ramps) graphically conceptually denote increase (left to right) or decrease (right to left) in the outcome specified within (basal metabolic rate (BMR)).

As previously mentioned, 80% of glucose clearance occurs via the muscle [14]. A decrease in muscle mass and muscle quality, as a result of sarcopenia, can lead to a series of issues leading to T2DM development. Decreased muscle quality results in decreased basal metabolic rate (BMR), which is defined as the minimum number of calories an organism requires to complete basic, life-sustaining functions. This number typically decreases when muscles deteriorate (quality/amount–mass). An individual’s body composition may change as a result of lifestyle factors, mainly diet and physical activity levels, leading to less muscle mass, hence decreased BMR and potentially increased fat mass, leading to obesity. Obesity has been strongly linked to T2DM development, where the peripheral cells grow resistant to the insulin produced by pancreatic β-cells. Because, largely, glucose clearance occurs by the muscle, decreased muscle quality and/or mass can lead to elevated inflammation—again, strongly associated with the induction of insulin resistance and the onset of T2DM. Finally, oxidative stress can lead to decreased muscle mass—again, inducing insulin resistance, although this pathway is not well understood currently. It is believed that an excess of reactive oxygen species form and transfer unpaired electrons, which then results in the oxidation of cellular machinery. In a healthy individual, antioxidants counter this process, but imbalance results in oxidative stress, glycation, and increased insulin resistance (Figure 1) [14,50,70,71].

To further illustrate the relationship between glucose clearance and muscle mass, Hong et al. [72] conducted a large-scale cohort study involving 113,913 men and 89,854 women, all free of T2DM at the beginning of the study. These individuals were then monitored using annual checkups over the course of 2.9 years on average. Skeletal muscle mass index was measured to indicate relative muscle mass changes. A total of 4264 individuals developed T2DM, and relative muscle mass was negatively associated with T2DM development, suggesting that muscle deterioration was associated with disease onset. It is important to note that the average age of these individuals was 39.1; therefore, even at a younger age, this association is still present [72].

A 2019 study aimed at examining the association between metabolic syndrome and sarcopenia observed 84 overweight or obese individuals (all over 50 years of age). These individuals were asked a series of lifestyle questions, including activity level, medical history, and demographics. Their waist circumference was measured, and they were then assessed for sarcopenia. A total of 52% of the individuals were considered obese, and 35% had severe sarcopenia prevalence. It was seen that metabolic syndrome is closely associated with greater lean muscle mass and forearm muscle size, but poorer overall muscle quality. Metabolic syndrome was also found to be negatively associated with all measures of muscle quality and positively associated with muscle mass and size, suggesting that increased muscle mass is not always an indicator of quality [73]. 

Scarce evidence is available regarding the benefit of common T2DM pharmacological therapies towards preventing or ameliorating sarcopenia among the elderly. Metformin, which is a typical first-line prescription pharmaceutical for improving insulin sensitivity in T2DM, has been shown to be associated with autophagic muscle cell death. Insulin stimulates muscle protein synthesis in the young, but not older age groups, thus not providing protection against age-related muscle decline and, finally, sarcopenia. Weight loss, which is a well-established and somewhat effective lifestyle modification to improve insulin sensitivity, may be associated with a reduction in lean body mass, contributing to sarcopenia, despite temporary improvements in the insulin sensitivity responses. Interestingly, the combination of amino acid supplementation regimes with vitamin D seem to benefit insulin sensitivity/T2DM and muscle mass/sarcopenia. Although the evidence is not abundant, muscle protein synthesis in response to protein supplementation in older adults may be enhanced by adequate vitamin D status. Daily supplementation of 2 g HMB, 5 g Arg and 1.5 g Lys for 12 months in older adults resulted in significant improvement in knee extension strength only for those whose baseline 25-hydroxy vitamin D test (25[OH]D) levels were ≥75 nmol/L [74]. Similarly, in older adults with sarcopenia, exercise plus daily whey protein (22 g), essential amino acids (11 g, including 4 g Leu) and vitamin D (100 IU) resulted in almost 2 kg greater gain in lean mass compared with exercise alone, as well as significant gains in hand grip strength and a decline in CRP levels [75].

Furthermore, Granic et al. (2019) observed that antioxidant and anti-inflammatory aspects of diet seem to enhance defending lean body mass, while simultaneously improving metabolic function and reducing the risk of insulin resistance and T2DM [76]. Oh et al. (2017) [77] evaluated protein intake in older Korean adults. Protein intake (<0.8 g protein/Kg/d), which was lower than the recommend level for adults, was associated with a higher risk of metabolic abnormalities in this study. In particular, a lower intake of protein contributed to a higher prevalence of metabolic risk factors in women than in men. More mechanistic metabolic studies in animal models showed that a mild decrease in essential amino acids, particularly cysteine, increased phosphorylation of eukaryotic initiation factor 2 alpha (eIF2α), thus inducing the integrated stress response (ISR) pathway leading to an overall adaptation that introduced the cell into a state of greater resilience in terms of metabolic abrogation [78]. Similar experiments in separate studies also revealed the indirect role of the mammalian target of rapamycin complex 1 (mTORC1), mediated by the amount of 4E-binding protein 1 (4E-BP1), again supporting the notion of a more resilient cell towards metabolic abrogation and, thus, metabolic syndrome [79]. Finally, recent developments have identified the microbiome as a key player/target for intervention, especially from a precision medicine perspective, for the potential risk modulation of metabolic syndrome and T2DM [70].

## 5. Conclusions

There is an evident relationship between sarcopenia and insulin resistance, and subsequently T2DM, where muscle mass degradation leads to decreased glucose clearance, resulting in increased insulin resistance and T2DM. There is also evidence suggesting that the addition of dietary supplements enriched in certain amino acids leads to improvements in muscle strength and muscle mass gain. The connections between sarcopenia, amino acid supplementation, and T2DM prevention has been supported by the literature, and evidence pointing towards a dietary supplement composed of HMB, Leu, Gln, and Arg is present, as highlighted in this review article. HMB and Leu have certainly been explored as a sarcopenia treatment, where they function to increase muscle protein synthesis. HMB has also been reported to have upregulated function when paired with Arg and Gln. Arg does not appear to prevent muscle deterioration alone, but when combined with HMB and Leu its effectiveness seems to be increased. Similar results have also been obtained with Gln alone and in combination with other amino acids. Therefore, no individual amino acid has been established to be adequate in preventing muscle deterioration, sarcopenia, and ultimately T2DM risk. However, in certain combinations, it does appear that a dietary supplement of the above-mentioned amino acids may stimulate an increase in muscle mass and muscle strength in sarcopenic elderly individuals, possibly decreasing T2DM risk, while mitigating sarcopenic effects. The optimal duration or dosing in an ideal mixture both remain important, as yet still unfulfilled, factors.

## Figures and Tables

**Table 1:** Summary of various amino acid supplementation regimes and their effects on health outcomes associated with sarcopenia.

Treatment	Inflammation Prevention	Increased Muscle Protein Synthesis	Reduced Muscle Deterioration	Increased Glucose Tolerance	Other Supplementation Required?	References
HMB	Yes	Yes	Yes	No	Yes	22, 23, 30, 31
Leu	No	Undetermined	No	Yes	Yes	32, 33, 34, 35, 36, 37, 38, 45
Gln	No	Yes	Yes	No	Yes	22, 23, 57
Arg	No	Yes	No	No	Yes	22, 23, 52, 54

## Abbreviations

WHO: World Health Organization, BMI: body mass index, HMB: hydroxyl-methyl butyrate, ARG: arginine, Leu: leucine, Gln: glutamine, SEAA: semi-essential amino acids, EAA: essential amino acids, GS: geriatric syndrome, EWGSOP: The European Working Group on Sarcopenia in Older People, IGF-1: insulin-like growth factor-1, TNF-α: tumor necrosis factor alpha, MuRF1: muscle ring finger 1, mTOR: mammalian target of rapamycin, USG: ultrasonography, BIA: bio-electrical impedance analysis, MRI: magnetic resonance imaging, DEXA: dual energy X-ray absorptiometry, CT: computerized tomography, SPPB: short physical performance battery, DHEA: dehydroepiandrosterone sulfate, GH: growth hormone, CNS: central nervous system, NRS: nutritional risk screening, SGA: subjective global assessment, MNA: mini nutritional evaluation, TTR: transthyretin, BCAA: branched chain amino acid, OBLA: occurrence of stable lactate accumulation, LBM: lean body mass.

## References

[1] Tayfur M. (2010). Yaşlı Diyabetik Erkeklerde Sarkopeni.

[2] Jong N. (2000). Nutrition and Senescence: Healthy Aging for All in the New Millennium. Nutrition.

[3] (2002). Diet, Nutrition and The Prevention of Chronic Diseases.

[4] World Health Organization (2005). Healthy Ageing. Practical Pointers on Keeping Well.

[5] van Abellan Kan G. (2009). Epidemiology and consequences of sarcopenia. J. Nutr. Health Aging.

[6] Fleg J.L., Lakatta E.G. (1988). Role of muscle loss in the age-associated reduction in VO2max. J. Appl. Physiol..

[7] Lindle R.S., Metter E.J., Lynch N.A., Fleg J.L., Fozard J.L., Tobin J., Roy T.A., Hurley B.F. (1997). Age and gender comparison of muscle strength in 654 women and men age 20–93 yr. J. Appl. Physiol..

[8] Rosenberg I.H. (1997). Sarcopenia: Origins and Clinical Relevance. J. Nutr..

[9] Santilli V., Bernetti A., Mangone M., Paoloni M. (2014). Clinical Definition of Sarcopenia. Clin. Cases Miner. Bone Metab. Off. J. Ital. Soc. Osteoporos. Miner. Metab. Skelet. Dis..

[10] Sieber C.C. (2019). Malnutrition and sarcopenia. Aging Clin. Exp. Res..

[11] Marcell T.J. (2003). Sarcopenia: Causes, consequences, and preventions. J. Gerontol. A Biol. Sci. Med. Sci..

[12] Öztürk Z.A. (2014). Tip II Diyabetes Mellituslu Sarkopenik Obez Kişilerde Kan Şekeri Regülasyonunun Sarkopeni Parametreleri Üzerine Etkisi.

[13] Boirie Y. (2009). Physiopathological mechanism of sarcopenia. J. Nutr. Heal. Aging.

[14] DeFronzo R.A., Devjit T. (2009). Skeletal Muscle Insulin Resistance ıs the Primary Defect in Type 2 Diabetes, Diabetes Care.

[15] Hickson M. (2015). Nutritional interventions in sarcopenia: A critical review. Proc. Nutr. Soc..

[16] Calvani R., Picca A., Marini F., Biancolillo A., Gervasoni J., Persichilli S., Primiano A., Coelho-Junior H.J., Bossola M., Urbani A. (2018). A Distinct Pattern of Circulating Amino Acids Characterizes Older Persons with Physical Frailty and Sarcopenia: Results from the BIOSPHERE Study. Nutrients.

[17] Candow D.G., Forbes S.C., Little J.P., Cornish S.M., Pinkoski C., Chilibeck P.D. (2012). Effect of nutritional interventions and resistance exercise on aging muscle mass and strength. Biogerontology.

[18] Wilson G.J., Wilson J.M., Manninen A.H. (2008). Effects of beta-hydroxy-beta-methylbutyrate (HMB) on exercise performance and body composition across varying levels of age, sex, and training experience: A review. Nutr. Metab..

[19] Noe J.E. (2009). L-Glutamine use in the treatment and prevention of mucositis and cachexia: A naturopathic perspective. Integr. Cancer Ther..

[20] Ogawa M., Yoshida N., Satomi-Kobayashi S., Tsuboi Y., Komaki K., Wakida K., Gotake Y., Inoue T., Tanaka H., Yamashita T. (2019). Efficacy of preoperative amino acid supplements on postoperative physical function and complications in open heart surgery patients: A study protocol for a randomized controlled trial. J. Cardiol..

[21] Clark R.H., Feleke G., Din M., Yasmin T., Singh G., Khan F.A., Rathmacher J.A. (2000). Nutritional treatment for acquired immunodeficiency virus-associated wasting using β-hyroxy-β-methylbutyrate, glutamine, and arginin: A randomized, double-blind, placebo-controlled study. J. Parenter. Enter. Nutr..

[22] Hsieh L.C., Chow C.J., Chang W.C., Liu T.H., Chang C.K. (2010). Effect of beta hydroxybeta methyl butyrate on protein metabolism in bed ridden elderly receiving tube feeding. Asia Pac. J. Clin. Nutr..

[23] May P.E., Barber A., D’Olimpio J.T. (2002). Reversal of cancer-related wasting using oral supplementation with a combination of β-hyroxy-β-methylbutyrate, arginine, and glutamine. Am. J. Surg..

[24] Mero A. (1999). Leucine Supplementation and Intensive Training. Sports Med..

[25] Berg J.M., Tymoczko J.L., Stryer L. (2002). Biochemistry.

[26] Fukagawa N.K. (2013). Protein and amino acid supplementation in older humans. Amino Acids.

[27] Wittmann F., Prix N., Mayr S., Angele P., Wichmann M.W., van den Engel N.K., Hernandez-Richter T., Chaudry I.H., Jauch K.W., Angele M.K. (2005). l-Arginine Improves Wound Healing after Trauma-Hemorrhage by Increasing Collagen Synthesis. J. Trauma Acute Care Surg..

[28] Williams J.Z., Abumrad N., Barbul A. (2002). Effect of a specialized amino acid mixture on human collagen deposition. Ann. Surg..

[29] Walrand S., Guillet C., Salles J., Cano N., Boirie Y. (2011). Physiopathological mechanism of sarcopenia. Clin. Geriatr. Med..

[30] Hsieh L.C., Chien S.L., Huang S., Tseng H.F., Chang C.K. (2006). Anti-inflammatory and anticatabolic effects of short-term β-hyroxy- β-methylbutyrate supplementation on chronic obstructive pulmonary disease patients in intensive care unit. Asia Pac. J. Clin. Nutr..

[31] Oktaviana J., Zanker J., Vogrin S., Duque G. (2018). The Effect of β-Hydroxy-β-Methylbutyrate (HMB) on Sarcopenia and Functional Frailty in Older Persons: A Systematic Review. J. Nutr. Heal. Aging.

[32] Katsanos C.S., Kobayashi H., Sheffield-Moore M., Aarsland A., Wolfe R.R. (2006). A high proportion of Leu is required for optimal stimulation of the rate of muscle protein synthesis by essential amino acids in the elderly. Am. J. Physiol. Metab..

[33] Koopman R., Verdijk L., Manders R.J.F., Gijsen A.P., Gorselink M., Pijpers E., Wagenmakers A.J., van Loon L.J.C. (2006). Co-ingestion of protein and Leucine stimulates muscle protein synthesis rates to the same extent in young and elderly lean men. Am. J. Clin. Nutr..

[34] Dardevet D., Sornet C., Balage M., Grizard J. (2000). Stimulation of in vitro rat muscle protein synthesis by Leucine decreases with age. J. Nutr..

[35] Rieu I., Sornet C., Bayle G., Prugnaud J., Pouyet C., Balage M., Papet I., Grizard J., Dardevet D. (2003). Leucine-supplemented meal feeding for ten days beneficially affects postprandial muscle protein synthesis in old rats. J. Nutr..

[36] Rieu I., Balage M., Sornet C., Giraudet C., Pujos E., Grizard J., Mosoni L., Dardevet D. (2006). Leucine supplementation improves muscle protein synthesis in elderly men independently of hyperaminoacidaemia. J. Physiol..

[37] Debras E., Prod’homme M., Rieu I., Balage M., Dardevet D., Grizard J. (2007). Postprandial Leucine deficiency failed to alter muscle protein synthesis in growing and adult rats. Nutrition.

[38] Yang Y., Breen L., Burd N., Hector A., Churchward-Venne T., Josse A., Tarnopolsky M.A., Phillips S. (2007). Resistance exercise enhances myofibrillar protein synthesis with graded intakes of whey protein in older men. Nutrition.

[39] Bell K.E., Snijders T., Zulyniak M., Kumbhare D., Parise G., Chabowski A., Phillips S.M. (2017). A whey protein-based multi-ingredient nutritional supplement stimulates gains in lean body mass and strength in healthy older men: A randomized controlled trial. PLOS ONE.

[40] Solerte S.B., Gazzaruso C., Bonacasa R., Rondanelli M., Zamboni M., Basso C., Locatelli E., Schifino N., Giustina A., Fioravanti M. (2008). Nutritional supplements with oral amino acid mixtures increases whole-body lean mass and insulin sensitivity in elderly subjects with sarcopenia. Am. J. Cardiol..

[41] Ferrando A.A., Paddon-Jones D., Hays N.P., Kortebein P., Ronsen O., Williams R.H., McComb A., Symons T.B., Wolfe R.R., Evans W. (2010). EAA supplementation to increase nitrogen intake improves muscle function during bed rest in the elderly. Clin. Nutr..

[42] Rondanelli M., Opizzi A., Antoniello N., Boschi F., Iadarola P., Pasini E., Aquilani R., Dioguardi F.S. (2011). Effect of amino acid supplementation on quality of life, amino acid profile and strength in institutionalized elderly patients. Clin. Nutr..

[43] Xu Z.E., Tan Z.J., Zhang Q., Gui Q.F., Yang Y.M. (2014). The effectiveness of Leucine on muscle protein synthesis, lean body mass and leg lean mass accretion in older people: A systematic review and meta-analysis. Br. J. Nutr..

[44] Murphy C.H., Saddler N.I., Devries M.C., McGlory C., Baker S.K., Phillips S.M. (2016). Leucine supplementation enhances integrative myofibrillar protein synthesis in free-living older men consuming lower- and higher-protein diets: A parallel-group crossover study. Am. J. Clin. Nutr..

[45] Backx E.M.P., Horstman A.M.H., Marzuca-Nassr G.N., van Kranenburg J., Smeets J.S., Fuchs C.J., Janssen A.A.W., de Groot L.C.P.G.M., Snijders T., Verdijk L.B. (2018). Leucine Supplementation Does Not Attenuate Skeletal Muscle Loss during Leg Immobilization in Healthy, Young Men. Nutrients.

[46] Trappe S., Creer A., Slivka D., Minchev K., Trappe T. (2007). Single muscle fiber function with concurrent exercise or nutrition countermeasures during 60 days of bed rest in women. J. Appl. Physiol..

[47] Trappe T.A., Burd N.A., Louis E.S., Lee G.A., Trappe S.W. (2007). Influence of concurrent exercise or nutrition countermeasures on thigh and calf muscle size and function during 60 days of bed rest in women. Acta Physiol..

[48] Trappe S., Creer A., Minchev K., Slivka D., Louis E., Luden N., Trappe T. (2008). Human soleus single muscle fiber function with exercise or nutrition countermeasures during 60 days of bed rest. J. Physiol. Integr. Comp. Physiol..

[49] Komar B., Schwingshackl L., Hoffmann G. (2014). Effects of Leucine-rich protein supplements on anthropometric parameter and muscle strength in the elderly: A systematic review and meta-analysis. J. Nutr. Heal. Aging.

[50] Cholewa J.M., Dardevet D., Lima-Soares F., de Araújo Pessôa K., Oliveira P.H., Dos Santos Pinho J.R., Nicastro H., Xia Z., Cabido C.E., Zanchi N.E. (2017). Dietary proteins and amino acids in the control of the muscle mass during immobilization and aging: Role of the MPS response. Amino Acids.

[51] Børsheim E., Bui Q.U., Tissier S., Kobayashi H., Ferrando A.A., Wolfe R.R. (2008). Effect of amino acid supplementation on muscle mass, strength and physical function in elderly. Clin. Nutr..

[52] Thalacker-Mercer A.E., Drummond M.J. (2014). The importance of dietary protein for muscle health in inactive, hospitalized older adults. Ann. N. Y. Acad. Sci..

[53] Souza M.K., Moraes M.R., Rosa T.S., Passos C.S., Neves R.V.P., Haro A.S., Cenedeze M.A., Arias S.C.A., Fujihara C.K., Teixeira S.A. (2019). l-Arginine Supplementation Blunts Resistance Exercise Improvement in Rats with Chronic Kidney Disease. Life Sci..

[54] Flakoll P., Sharp R., Baier S., Levenhagen D., Carr C., Nissen S. (2004). Effect of β-hydroxy-β-methylbutyrate, arginine, and lysine supplementation on strength, functionality, body composition, and protein metabolism in elderly women. Nutrition.

[55] Haba Y., Fujimura T., Oyama K., Kinoshita J., Miyashita T., Fushida S., Harada S., Ohta T. (2019). Effect of Oral Branched-Chain Amino Acids and Glutamine Supplementation on Skeletal Muscle Atrophy After Total Gastrectomy in Rat Model. J. Surg. Res..

[56] Mignon M., Lêvêque L., Bonnel E., Meynial-Denis D. (2007). Does Glutamine Supplementation Decrease the Response of Muscle Glutamine Synthesis to Fasting in Muscle in Adult and Very Old Rats?. J. Parenter. Enter. Nutr..

[57] Meynial-Denis D., Patureau Mirand P. (2011). Is Glutamine the Cornerstone of Sarcopenia in Very Old Individuals?. J. Nutr. Health Aging.

[58] Tatti P., Barber A.E. (2012). The use of a specialized nutritional supplement for diabetic foot ulcers reduces the use of antibiotics. J. Endocrinol. Metab..

[59] Baier S., Johannsen D., Abumrad N., Rathmacher J.A., Nissen S., Flakoll P. (2009). Year-long changes in protein metabolism in elderly men and women supplemented with a nutrition cocktail of betahydroxy-beta-methylbutyrate (HMB), L-arginine, and L-lysine. JPEN.

[60] Olza J., Mesa M.D., Poyatos R.M., Aguilera C.M., Moreno-Torres R., Perez de la Cruz A., Gil A. (2010). A specific protein-enriched enteral formula decreases cortisolemia and improves plasma albumin and amino acid concentrations in elderly patients. Nutr. Metab..

[61] Cruz-Jentoft A.J., Baeyens J.P., Bauer J.M., Boirie Y., Cederholm T., Landi F., Martin F.C., Michel J.-P., Rolland Y., Schneider S.M. (2010). European Working Group on Sarcopenia in Older People. Sarcopenia: European consensus on definition and diagnosis: Report of the European Working Group on Sarcopenia in Older People. Age Ageing.

[62] Nissen S., Sharp R., Ray M., Rathmacher J.A., Rice D., Fuller J.C., Connelly A.S., Abumrad N. (1996). Effect of Leucine metabolite β-hydroxy-β-methylbutyrate on muscle metabolism during resistance-exercise training. J. Appl. Physiol..

[63] Nissen S.L., Sharp R.L. (2003). Effect of dietary supplements on lean mass and strength gains with resistance exercise: A meta-analysis. J. Appl. Physiol..

[64] Marzani B., Balage M., Vénien A., Astruc T., Papet I., Dardevet D., Mosoni L. (2008). Antioxidant supplementation restores defective leucine stimulation of protein synthesis in skeletal muscle from old rats. J. Nutr..

[65] Garlick P.J., Grant I. (1988). Amino acid infusion increases the sensitivity of muscle protein synthesis in vivo to insulin. Effect of branched-chain amino acids. Biochem. J..

[66] Stancliffe R.A. (2012). Role of beta-hydroxy-beta-methylbutyrate (HMB) in Leucine stimulation of mitochondrial biogenesis and fatty acid oxidation. FASEB J..

[67] Rathmacher J., Nissen S., Panton L., Clark R., May P., Barber A., D’Olimpio J., Abumrad N. (2004). Supplementation with a combination of beta-hydroxy-beta-methylbutyrate (HMB), arginine, and glutamine is safe and could improve hematological parameters. J. Parenter. Enter. Nutr..

[68] Calvani R., Miccheli A., Landi F., Bossola M., Cesari M., Leeuwenburgh C., Sieber C.C., Bernabei R., Marzetti E. (2013). Current nutritional recommendations and novel dietary strategies to manage sarcopenia. J. Frailty Aging.

[69] Johnson C.D. Nutrition, Muscle Mass, and Muscular Performance in Middle Age and Beyond. Proceedings of the The 110th Abbott Nutrition Research Conference.

[70] Sikalidis A.K., Maykish A. (2020). The Gut Microbiome and Type 2 Diabetes Mellitus: Discussing a Complex Relationship. Biomedicines.

[71] Betteridge D.J. (2000). What is oxidative stress?. Metab. Clin. Exp..

[72] Hong S., Chang Y., Jung H.S., Yun K.E., Shin H., Ryu S. (2017). Relative muscle mass and the risk of incident type 2 diabetes: A cohort study. PLOS ONE.

[73] Mesinovic J., McMillan L.B., Shore-Lorenti C., De Courten B., Ebeling P.R., Scott D. (2019). Metabolic Syndrome and Its Associations with Components of Sarcopenia in Overweight and Obese Older Adults. J. Clin. Med..

[74] Clavel S. (2006). Atrophy-related ubiquıtın ligases, Atrogin-1 and MuRF1 are upregulated in aged rat tibialis anterior muscle. Mech. Ageing Dev..

[75] Scott D., de Courten B., Ebeling P.R. (2016). Sarcopenia: A potential cause and consequence of type 2 diabetes in Australia’s ageing population?. Med. J. Aust..

[76] Granic A., Sayer A.A., Robinson S.M. (2019). Dietary Patterns, Skeletal Muscle Health, and Sarcopenia in Older Adults. Nutrients.

[77] Oh C., No J. (2017). Does Protein Intake Affect Metabolic Risk Factors among Older Adults in Korea?. J. Obes. Metab. Syndr..

[78] Sikalidis A.K., Stipanuk M.H. (2010). Growing rats respond to a sulfur amino acid-deficient diet by phosphorylation of eIF2α and induction of adaptive components of the integrated stress. J. Nutr..

[79] Sikalidis A.K., Mazor K.M., Kang M., Liu H., Stipanuk M.H. (2013). Total 4E-BP1 Is Elevated in Liver of Rats in Response to Low Sulfur Amino Acid Intake. J. Amino Acids.

